# Landet: an efficient physics-informed deep learning approach for automatic detection of anatomical landmarks and measurement of spinopelvic alignment

**DOI:** 10.1186/s13018-024-04654-7

**Published:** 2024-03-25

**Authors:** AliAsghar MohammadiNasrabadi, Gemah Moammer, Ahmed Quateen, Kunal Bhanot, John McPhee

**Affiliations:** 1 Department of Systems Design Engineering, University of Waterloo, 200 University Ave W, Waterloo, ON N2L 3G1 Canada; 2 Department of Spine Surgery, Grand River Hospital (GRH), 835 King St W, Kitchener, ON N2G 1G3 Canada; 3 Department of Surgery, McMaster University, 1280 Main Street West, Hamilton, ON L8S 4K1 Canada

**Keywords:** Automatic Landmark Detection, Spinopelvic Parameters, Deep Learning, Physics-informed Constraints

## Abstract

**Purpose:**

An efficient physics-informed deep learning approach for extracting spinopelvic measures from X-ray images is introduced and its performance is evaluated against manual annotations.

**Methods:**

Two datasets, comprising a total of 1470 images, were collected to evaluate the model’s performance. We propose a novel method of detecting landmarks as objects, incorporating their relationships as constraints (***LanDet***). Using this approach, we trained our deep learning model to extract five spine and pelvis measures: Sacrum Slope (SS), Pelvic Tilt (PT), Pelvic Incidence (PI), Lumbar Lordosis (LL), and Sagittal Vertical Axis (SVA). The results were compared to manually labelled test dataset (GT) as well as measures annotated separately by three surgeons.

**Results:**

The ***LanDet*** model was evaluated on the two datasets separately and on an extended dataset combining both. The final accuracy for each measure is reported in terms of Mean Absolute Error (MAE), Standard Deviation (SD), and R Pearson correlation coefficient as follows: $[SS^\circ : 3.7 (2.7), R = 0.89]$, $[PT^\circ : 1.3 (1.1), R = 0.98], [PI^\circ : 4.2 (3.1), R = 0.93], [LL^\circ : 5.1 (6.4), R=0.83], [SVA(mm): 2.1 (1.9), R = 0.96]$. To assess model reliability and compare it against surgeons, the intraclass correlation coefficient (ICC) metric is used. The model demonstrated better consistency with surgeons with all values over 0.88 compared to what was previously reported in the literature.

**Conclusion:**

The ***LanDet*** model exhibits competitive performance compared to existing literature. The effectiveness of the physics-informed constraint method, utilized in our landmark detection as object algorithm, is highlighted. Furthermore, we addressed the limitations of heatmap-based methods for anatomical landmark detection and tackled issues related to mis-identifying of similar or adjacent landmarks instead of intended landmark using this novel approach.

## Introduction

The assessment and prediction of the geometric characteristics of the spinopelvic complex have garnered significant interest among both the clinical and research communities [1–3]. Radiological examination of the spine and pelvis plays a crucial role in both surgical and non-surgical treatments of spinal disorders [4]. Understanding the sagittal balance of the spine and pelvis, which entails the interplay between spine and pelvic measures, is crucial for maintaining postural equilibrium. Initially, measurements of sagittal balance were conducted manually using conventional radiographs and later assisted by computer-based tools [5]. However, the inherent limitations of radiographic imaging and subjectivity in manual measurements introduced errors [6].

To address these challenges, machine learning techniques, a subset of artificial intelligence (AI), were employed, allowing computer models to recognize patterns in data [7]. With the advancement of deep learning (DL), a specialized branch of machine learning that emulates the information processing of neural systems, performance in automated image analysis significantly improved. DL methods excel in learning optimal features and feature compositions without human-designed feature extraction. Consequently, DL has found extensive application in various domains, including radiology, musculoskeletal radiology, and spinal disorders [1].

The concept of sagittal spinopelvic balance has gained widespread recognition among radiologists and spine professionals, as it is essential for understanding the etiopathogenesis of spinal deformities and selecting appropriate treatment options [5]. Evaluating sagittal balance typically involves radiographic measurements of geometric relationships among specific anatomical landmarks in sagittal X-ray images. Measures such as Sacral Slope (SS), Pelvic Tilt (PT), Pelvic Incidence (PI), Lumbar Lordosis (LL), and Sagittal Vertical Axis (SVA) are commonly used to assess sagittal balance [8]. However, manual measurements can be subjective, time-consuming, and prone to inaccuracies due to the complexity of accurately identifying anatomical landmarks. To overcome these challenges, various computer-assisted tools and software have been developed, but they still rely on observer input [9].

Recent advancements in artificial intelligence and deep learning techniques have provided promising avenues for automating the process of extracting anatomical parameters from radiographic images of the spine [10]. These technologies offer the potential to enhance efficiency, reduce the burden on healthcare professionals, and improve the accuracy of measurements [11]. Additionally, they enable the analysis of a wide range of clinical scenarios, including sagittal and coronal deformities, degenerative phenomena, and images acquired with varying fields of view [10].

In this context, several studies have proposed innovative methods leveraging deep learning algorithms to automate the measurement of spinal alignment parameters. These approaches encompass techniques ranging from fully convolutional neural networks with differentiable spatial to numerical layers (DSNT) to fine-tuned Mask R-CNN models for vertebral segmentation [10, 12]. The results of these studies have shown promising outcomes, with accurate predictions of vertebral locations and sagittal alignment parameters, despite inherent challenges and limitations [12, 13].

While these automated methods demonstrate significant potential, it is essential to critically assess their performance and address the associated challenges. These include potential variations in image quality, noise, and the presence of different spinal pathologies [12, 13]. Moreover, it is imperative to evaluate the reliability and reproducibility of automated measurements in comparison to manual assessments performed by experienced clinicians [5].

Heatmap-based regression, the most common method used for landmark detection in recent studies [5, 13–15], has some inherent drawbacks including issues with overlapping heatmap signals and post-processing requirements. We are thus motivated to introduce a new approach in which landmarks are considered as objects using bounding boxes. Certain anatomical landmarks are used to extract spinopelvic measures from lateral X-ray images and geometric constraints are imposed to the model to improve the predictions. In addition, previous models sometime may fail to distinguish between adjacent and similar anatomical landmarks [13]. We have addressed this issue by introducing a novel geometrical constraint physics-informed model. This novel deep learning model not only detects each anatomical landmark as an unique object, but also creates a graph of relationship between objects, which helps the model to locate the landmarks more accurately.

## Materials and methods

### Spinopelvic geometric measurements

**Fig. 1:**
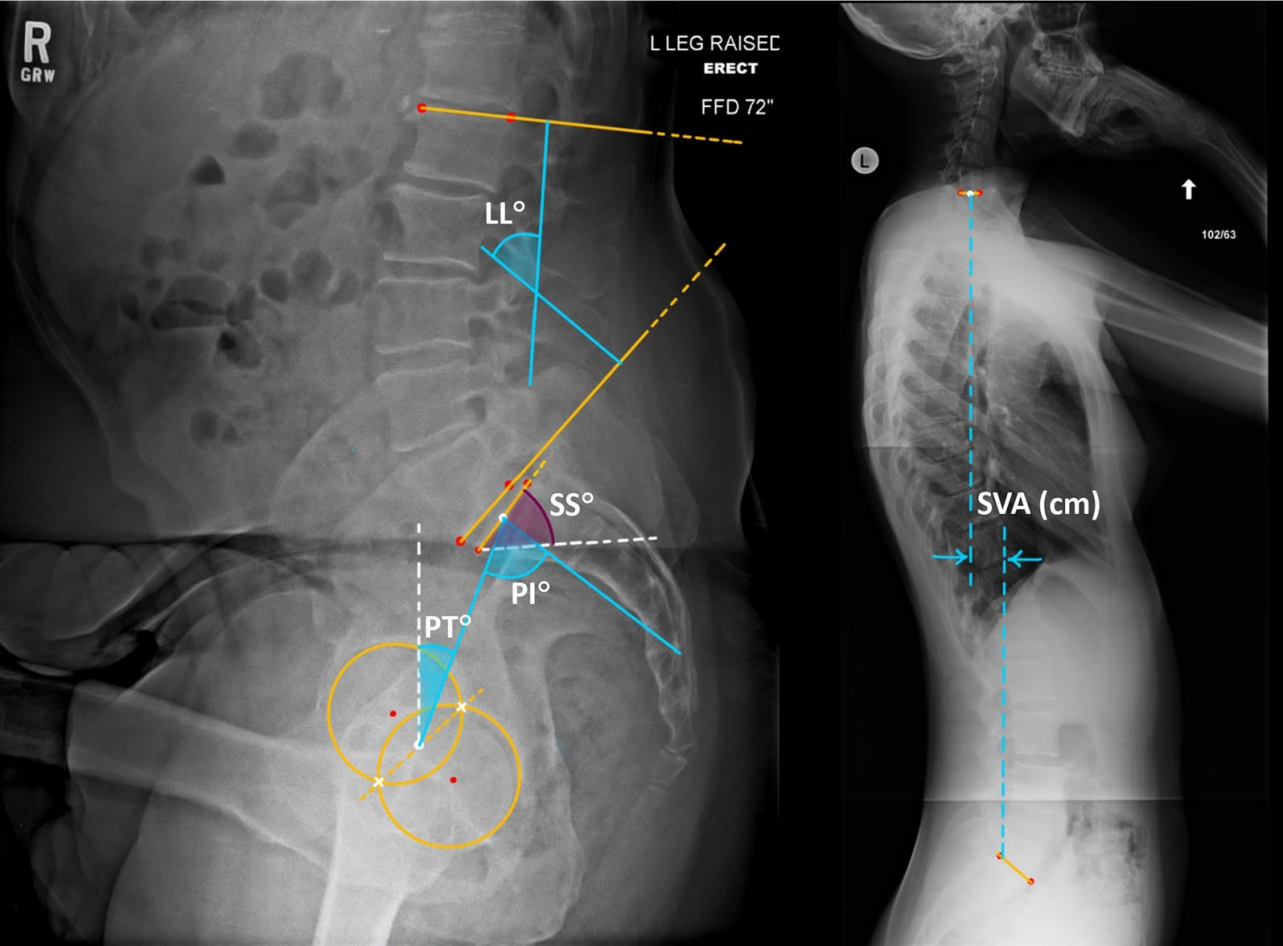
Main spinopelvic measures in lateral X-ray images, considering both femoral heads to calculate the center of rotation for the pelvis

In this research we targetted five anatomical measures to be extracted automatically, which are Sacrum Slope (SS), Pelvic Tilt (PT), Pelvic Incidence (PI), Lumbar Lordosis (LL) and Sagittal Vertical Axis (SVA). As the Fig. 1 shows, SS is the angle between the tangent line to the upper end plate of S1 (connecting line of posterior and anterior corners) and the horizontal reference line. PT is the angle between the vertical reference line and the connecting line between the midpoint of sacrum upper end plate and the midpoint of connecting line between the intersection of femoral head circles (hip axis). Pelvic Incidence (PI) is a morphologic measure defined as the angle between the perpendicular line to the mid-point of the upper end plate of last vertebra of the sacrum (S1) and the connecting line of this point to the midpoint of connecting line between the intersection of femoral head circles [16]. This measure is defined as an anatomical measure and strictly related to the shape of the pelvis [17]. This measure mostly remains constant in each pelvic posture and it is equal to the sum of two measures (PT and SS) [18]. Lumbar Lordosis (LL) is determined by the angle between two lines: one connecting the two inferior corners of the L5 vertebra’s plate and the other connecting the two superior corners of the L1 vertebra’s plate. The Sagittal Vertical Axis (SVA) measures spinopelvic alignment and is determined by the horizontal distance between the upper posterior corner of the sacrum and the midpoint of the C7 vertebra.

### Dataset and data preparation

We collected a total of 1,150 lateral spine X-ray images (DS1) from patients referred to Grand River Hospital (GRH) in Kitchener, Canada, between 2016 and 2022. Additionally, we incorporated a dataset (DS2) of 320 lateral lumbar spine and pelvic images provided by Intellijoint Surgical. Our datasets encompasses a comprehensive range of cases, including patients with hip or spine implants and images from both sitting and standing postures. Unlike some other research [5, 13], we included all images, even those with poor contrast or partial spine visibility. To address these issues, we employed appropriate image processing filters to enhance annotation accuracy for parts with high or low intensity. By including partial spine images, our dataset enables the model to identify anatomical landmarks effectively, even in incomplete images. The utilization of two distinct datasets enabled us to evaluate the model’s performance on different data sizes and imaging systems. DS1 consisted of images captured using ordinary X-ray devices, while DS2 utilized the EOS imaging system. To facilitate training, validation, and testing, we divided the datasets into sets comprising 80%, 10%, and 10% of the total data, respectively. We note the uniqueness of our dataset where each X-ray image corresponds to a distinct patient case to ensure a wide representation of clinical scenarios. However, an exception exists within Dataset 2 (DS2), which comprises 50 patients each represented by both sitting and standing X-ray images. To mitigate the risk of overfitting our model, these dual-position images were deliberately excluded from our test dataset.

To address the challenges posed by the limited size of our datasets, we implemented a data augmentation strategy to enhance the diversity of our training samples, thereby improving model detection accuracy. Specifically, our augmentation techniques included $\left( 1 \right)$ cropping, to simulate the effect of partial object occlusion and introduce variability in object positioning, $\left( 2 \right)$ flipping, to ensure the model’s robustness to changes in object orientation, $\left( 3 \right)$ mosaic augmentation, a technique that combines several images to create a single training sample with a mosaic-like appearance. This helps the model learn to detect objects in complicated scenes where objects may overlap, and $\left( 4 \right)$ rotation, to accustom the model to recognizing objects at different angles.

**Fig. 2:**
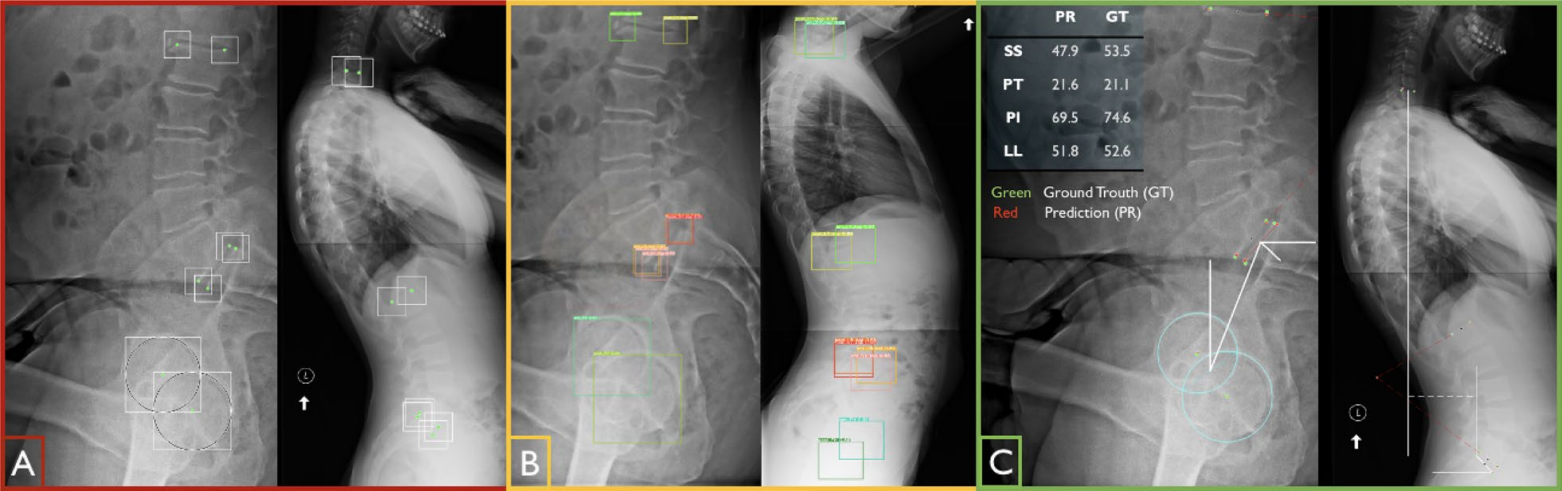
**A** Manual labeling of landmarks and specifying bounding boxes. **B** Extracted landmarks as objects with different confidences, trained model output, evaluated with the test set. **C** choosing the highest confidence for each landmark as the prediction results and calculation of the spinopelvic measures

A Matlab graphical user interface (GUI) was developed to facilitate image annotation. In this process, 10 anatomical landmarks are manually annotated by the researchers in each image using the GUI. The GUI allows annotators to identify the desired points, which automatically generates corresponding bounding boxes. For accurate femoral head annotations, annotators are required to specify three points on the edge of each femoral head. The GUI then calculates and visualizes the center of the femoral heads based on the specified points. The sizes of the bounding boxes are optimized for the vertebrae, set at 5% of the maximum image size. However, for the femoral heads, the bounding box sizes vary depending on the size of the femoral head circles. To ensure consistency, the coordinates and bounding box sizes are normalized to the maximum dimension of the image, resulting in coordinate values ranging from 0 to 1. The resulting labels are shown in Fig. 2A.

### Overview on landmark detection using landmarks as constraint objects

To automatically extract spinopelvic measures of interest (such as SS, PT, PI, LL, and SVA), we adopt a landmark detection approach that treats landmarks as objects. Our method utilizes a deep learning physics-based object detection algorithm, which overcomes limitations of heatmap-based regression methods, including issues with overlapping heatmap signals and post-processing requirements [19]. In our approach, landmarks are represented as objects with bounding boxes centered at the landmark coordinates (bx, by) and equal width (bw) and height (bh). Our labeled dataset comprises 10 classes of landmark objects ($c_i$), including the centers of femoral heads and the anterior/posterior points of S1, L1, C7 superior end plates, and L5 inferior end plate. Each label includes the class number and the bounding box features $C_i = (c_i, bx_i, by_i, bw_i, bh_i)$. Our deep learning model predicts objects with varying confidence levels (Fig. 2B), and the object with the highest confidence is used to extract the desired landmarks. To elucidate the rationale for considering constraints between these anatomical landmarks, we can consider the scenario where a surgeon intends to annotate a lateral X-ray image in order to measure the SS. When the posterior corner of the superior sacrum end plate is identified, the surgeon can leverage the predictable relationship within the sacrum as a solid body to determine the corresponding anterior point. We refer to this relationship as a geometric constraint, and the same concept has been applied for other landmarks. Imposing these constraints helps the model not only understand the features of each object (landmarks) but also enables a holistic understanding of the entire image and the interrelationships (constraints) among these landmarks. The final detected landmarks are subsequently used to calculate the desired measurements (Fig. 2C).

### Model architecture

In an effort to address the aforementioned limitations and drawbacks of heatmap-based regression method (e.g., quantization error, overlapping heatmap signals, and high computational requirements [19]), and to provide a more efficient alternative, we introduce a novel approach called ***LanDet*** (**Land**mark **Det**ection). Instead of relying on heatmaps, ***LanDet*** models individual landmarks as objects within a dense single-stage anchor-based detection framework. Furthermore, the relations between landmarks are imposed to the detection architecture as geometrical constraints. This innovative method aims to improve the efficiency and accuracy of anatomical landmark detection and clinical measurements without the need for heatmaps.

**Fig. 3:**
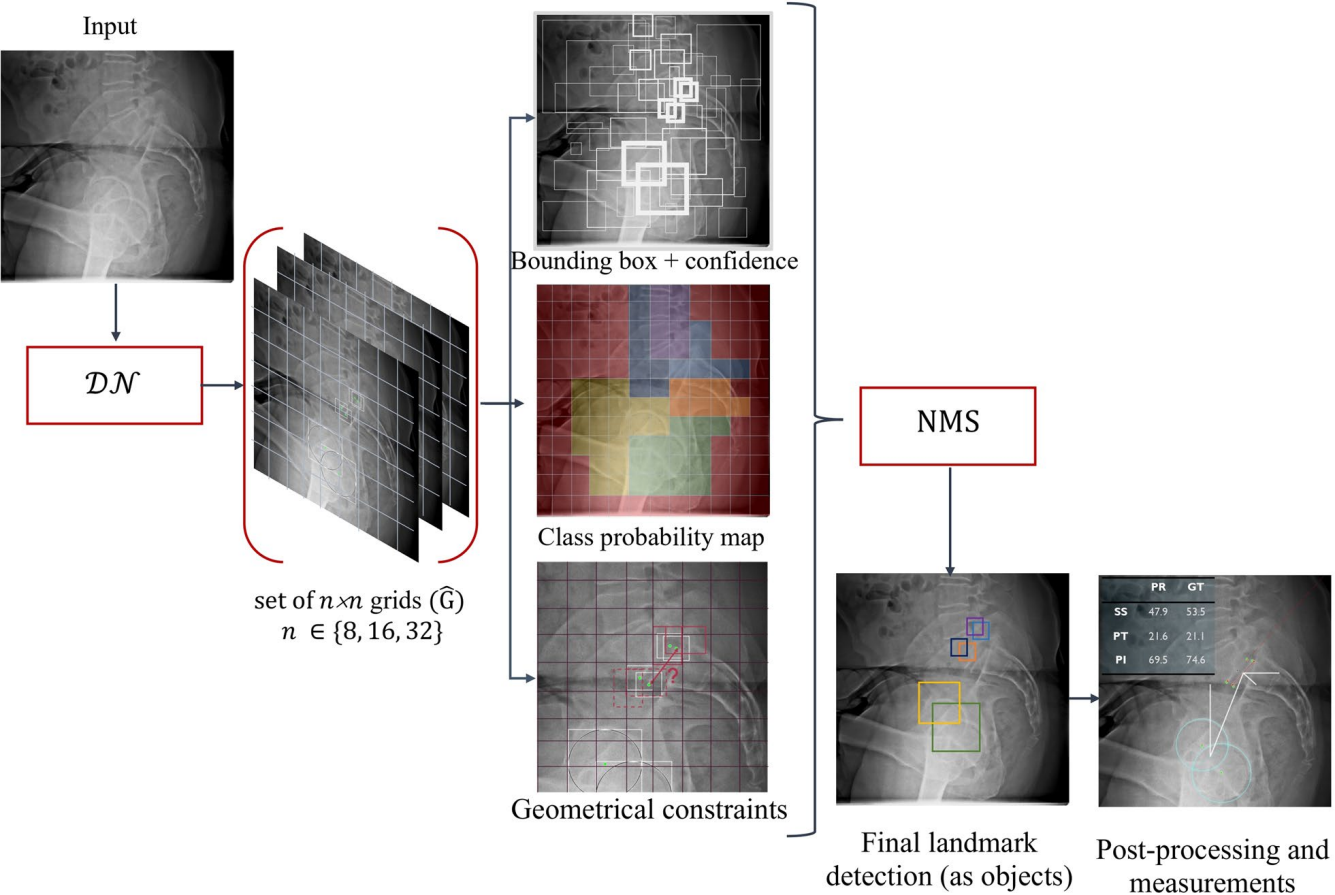
***LanDet*** utilizes a dense detection network denoted as *DN* that is trained with the multi-task loss $LanDet_{loss}$. The purpose of this network is to transform an input image represented by *I* into a collection of output grids denoted as ${\hat{G}}$. These grids contain the predicted landmark objects represented by ${\hat{O}}_l$. To obtain potential detection, a technique called non-maximum suppression (NMS) [19] is employed for ${\hat{O}}_l$. The geometrical constraints are applied on these candidate detections to generate the final predictions for ${\hat{O}}_l$, which then are used to calculate desired measures

As can be seen in Fig.  3, our model works with splitting the image into grid cells and each cell is responsible for predicting bounding boxes, by evaluating whether or not the center of the bounding box lies in the cell. Anchor boxes enable the model to predict more than one object in a single cell. Figure 3 displays a diagram illustrating the ***LanDet*** pipeline. This pipeline utilizes a deep convolutional neural network denoted as *DN*, which takes an input image *I* with dimensions $h \times w \times 3$ and transforms it into a collection of three output grids denoted as ${\hat{G}}$. These grids contain the predicted objects denoted as ${\hat{O}}$. Each individual grid, denoted as ${\hat{G}}_n$, has dimensions $\frac{h}{n} \times \frac{w}{n} \times N_a \times N_o$, where *n* takes on values from the set $\{8, 16, 32\}$. The transformation performed by the deep network can be expressed as the following equation:

1 $$\begin{aligned} DN(I) = {\hat{G}} \end{aligned}$$

$N_a$ represents the count of anchor channels, while $N_o$ corresponds to the number of output channels for each object. The feature extractor *DN* follows the YOLO-style approach [20] and makes effective use of Cross-Stage-Partial (CSP) bottlenecks [19, 21].

Due to the properties of strided convolutions, the characteristics of an output grid cell ${{\hat{G}}^{i,j}}_n$ are influenced by the image patch $I_p$, defined as $I_{n_i:n_{i+1},n_j:n{j+1}}$. Consequently, if the center of a target object $(b_x, b_y)$ lies within $I_p$, the corresponding output grid cell ${{\hat{G}}^{i,j}}_n$ is responsible for detecting that object. The effective range of perception of an output grid expands as *n* increases, implying that smaller output grids are more effective at detecting larger objects. The boounding box sizes are set to be 5% of the maximum size of the image for the landmarks on the spinal part, and for the femoral heads, the bounding box sizes are equal to the diameter of femoral cup so that it can enclose that part.

The output grid cells ${{\hat{G}}^{i,j}}_n$ encompass $N_a$ anchor channels, which are associated with anchor boxes $A_n = {{\{(A_{w_a}, A_{h_a})}\}^{N_a}_{a=1}}$. To assign a target object *O* to an anchor channel, a tolerance-based matching of their respective sizes is employed. This approach introduces redundancy, allowing the grid cells ${{\hat{G}}^{i,j}}_n$ to detect multiple objects and specialize in detecting objects of various sizes and shapes. Moreover, additional redundancy in detection is achieved by enabling the neighboring grid cells ${{\hat{G}}_n}^{{i\pm 1,j}}$ and ${{\hat{G}}_n}^{{i,j\pm 1}}$ to detect objects within the same image patch $I_p$ [22, 23].

#### Loss function

To introduce the relations between each landmark to the model, we modified the main object detection loss function to incorporate the geometric constraints. A set of target grids *G* is created, and a multi-task loss function $LanDet_{loss}$ is employed to train the model to learn various aspects, including the objectness ${\hat{p}}_o$ (represented by $l_{\text {obj}}$), the intermediate bounding boxes ${\hat{t}}$ ($l_{\text {box}}$), the class scores ${\hat{c}}$ ($l_{\text {cls}}$), and the intermediate constraint satisfaction ${\hat{r}}$ ($l_{\text {cnst}}$). To compute the loss components for a single image, the following procedure is followed:

2 $$\begin{aligned} {LanDet}_{loss}\ =l_{box}\ \ +\ l_{obj}\ \ +\ l_{cls}\ \ +\sum _{i=1}^{k}{w_i.l_{cnst}} \end{aligned}$$

where *k* is the number of defined constraints and $w_i$ are the weights for each of the constraints. Other parts of this loss function are defined as followed:

3 $$\begin{aligned} l_{\text {obj}}&= \sum _{n} \frac{1}{\text {num}(G_n)} \sum _{G_n} \text {BCE}({\hat{p}}_o, p_o \cdot \text {IoU}({\hat{t}}, t)) \end{aligned}$$

4 $$\begin{aligned} l_{\text {box}}&= \sum _{n} \frac{1}{\text {num}(O \in G_n)} \sum _{O \in G_n} (1 - \text {IoU}({\hat{t}}, t)) \end{aligned}$$

5 $$\begin{aligned} l_{\text {cls}}&= \sum _{n} \frac{1}{\text {num}(O \in G_n)} \sum _{O \in G_n} \text {BCE}({\hat{c}}, c) \end{aligned}$$

6 $$\begin{aligned} l_{\text {cnst}}&= \sum _{n} \frac{1}{\text {num}(O \in G_n)} \sum _{i \in O_{l_i}} \sum _{j \in O_{l_j}} f_{c}({\hat{r}}_{ij}, r_{i_j}) \end{aligned}$$

where BCE represents “binary cross-entropy”, and “intersection over union” known as IoU (measures the overlap between the predicted bounding box and the ground truth bounding box [24]), are crucial elements and defined as follows:

7 $$\begin{aligned} \text {BCE}(c, {\hat{c}})&= - \left( c \cdot \log ({\hat{c}}) + (1 - c) \cdot \log (1 - {\hat{c}}) \right) \end{aligned}$$

8 $$\begin{aligned} \text {IoU}&= \frac{\text {Area of Intersection}}{\text {Area of Union}} \end{aligned}$$

Additionally, $f_c$ represents a regression-based function, which defines the correlation between landmarks. For any angular constraint, $f_c$ represents a cosine similarity function and for the distance constraints, $f_c$ represents the absolute distance loss. To include $f_c$ term in the cost function, *r* is defined as vectors and distances between all pairs of the 10 anatomical landmarks in the angular and distance constraints, respectively. When $G_n^{i,j,a}$ represents a target object *O*, the value of the target objectness $p_o$ is determined by multiplying it with the IoU score to encourage specialization within the anchor channel predictions [20]. Conversely, when $G_n^{i,j,a}$ does not represent a target object, $p_o$ is set to 0. Practical implementation involves applying the losses to a batch of images using batched grids. The total loss $LanDet_{loss}$ is computed as a weighted sum of the loss components, scaled by the batch size $n_b$:

9 $$\begin{aligned} {LanDet}_{loss} = n_b\left(\lambda _{\text {obj}}l_{\text {obj}} + \lambda _{\text {box}}l_{\text {box}} + \lambda _{\text {cls}}l_{\text {cls}} + \lambda _{\text {cnst}}\sum _{i=1}^{k}{w_i.l_{cnst}}\right) \end{aligned}$$

where each $\lambda$ is the weight for the corresponding loss measurement.

### Model measurement performance and evaluation metrics

To evaluate landmark detection as objects, mean Average Precision (mAP) was used. The calculation of mAP involves several metrics and components, including intersection over union (IOU), precision, recall, precision-recall curve, and average precision (AP). To assess the accuracy of the model predictions, we employ the relative root mean square error (RRMSE) to compare the predicted values (PR) with the ground truth labels (GT). The RRMSE is computed using the following equation:

10 $$\begin{aligned} \text {RRMSE} = \frac{ \sqrt{\frac{1}{N} \sum _{i=1}^{N} (y_i - v_i)^2}}{\sqrt{\frac{1}{N} \sum _{i=1}^{N} (y_i - {\bar{y}})^2}} \end{aligned}$$

Here, $y_i$ represents the measured quantity we aim to predict, $v_i$ denotes our model’s prediction, and ${\bar{y}}$ is the mean of the ground truth labels, defined as ${\bar{y}} = \frac{1}{N} \sum _{i=1}^{N} y_i$. The RRMSE is a dimensionless metric, where a lower value indicates better accuracy (0 being the optimal value and 1 representing the threshold of uselessness). Additionally, we define the detection accuracy as:

11 $$\begin{aligned} \text {Accuracy} = (1 - \text {RRMSE}) \times 100 \end{aligned}$$

To evaluate the consistency among surgeons’ annotations and the quality of the ground truth labeling, we involved three senior surgeons to review and annotate the test dataset. We calculated the intraclass correlation coefficient (ICC) between each reviewer, as well as between the GT and PR measurements. This analysis helps us assess the level of agreement and inconsistencies in the annotations. We have also evaluated the model reliability by comparing the surgeons’ measurements with model prediction using the ICC metric. The ICC is a measure used to assess the reliability or agreement among surgeons, GT, and PR in this study. As suggested in [25], we have used the single-rating consistency model as follows:

12 $$\begin{aligned} ICC = \frac{{MSBS - MSWS}}{{MSBS + (m-1)\; MSWS}} \end{aligned}$$

where MSBS: represents the Mean Square Between Subjects (variance between annotators), MSWS: represents the Mean Square Within Subjects (variance within annotators), m: represents the number of annotators. Using this metric, we can evaluate the model’s reliability by examining the ICC value for each measure in our comparison.

## Results and discussion

In this section, we present the results and discuss the performance of the ***LanDet*** model on the test datasets, which consisted of 140 images. The model successfully detected all landmarks in 137 images, achieving an overall detection rate of 98%. However, it encountered difficulties in two images where it failed to identify the femoral heads and one image where the sacrum landmarks were missed. Note that during manual annotation, the annotators also faced challenges in identifying femoral heads in six test images due to obstacles or partial image cutoffs in that specific area. The model predicted the location of femoral heads in these challenging cases, demonstrating its robustness. Moreover, the model showed excellent performance in detecting landmarks, even in scenarios involving spinal or hip implants and low-quality images, despite the limited data available for these cases in the training and validation datasets. We will further discuss the accuracy of the model’s landmark detection in the following sections.

### Model performance and accuracy of automated measurements

We assessed the performance of the ***LanDet*** model using two separate datasets and combined them into an extended dataset. The inclusion of these datasets separately allowed us to examine the model’s performance on different-sized datasets (DS1: 1150 images and DS2: 320 images) and evaluate its performance on two types of X-ray images from different devices (DS1: ordinary and DS2: EOS). The IoU threshold is set to be 0.3 for the predicted bounding boxes to be considered successful detections. Table 1 presents the results of the ***LanDet*** model’s performance, including the average error, mean and standard deviation of predicted values, mean and standard deviation of ground truth data, and the accuracy of the model’s predictions. The table reveals that SVA and PT measures demonstrated better accuracy in DS1, with the model achieving 92.8% and 91.1% accuracy, respectively. In DS2, PT and PI measures exhibited the highest accuracy, with 89.2% and 86.6%, respectively. Notably, the model demonstrated good performance even in the dataset with a limited number of images (DS2), particularly in the prediction of the PT measure. While increasing the number of images in the dataset led to improved detection performance, the model’s performance remained commendable in DS2.

**Table 1:** Statistical comparison of the values of reference manual parameter measurements (GT) and those obtained automatically by the prediction model (PR)

	MAE		Mean PR (±SD)		Mean GT (±SD)		Acc (%)	
	DS1	DS2	DS1	DS2	DS1	DS2	*DS1*	*DS2*
SS^∘^	4.8	6.2	42.5 (8.7)	34.9 (11.8)	38.9 (10.3)	30.5 (10.4)	*90.2*	*84.7*
PT^∘^	1.8	2.4	18.1 (10.6)	21.7 (21.4)	19.3 (9.4)	22.1 (19.5)	*91.1*	***89.2***
PI^∘^	5.7	7.9	53.6 (10.2)	62.4 (10.7)	50.8 (12.6)	56.7 (13.7)	*90.1*	*86.6*
LL^∘^	6.7	7.5	48.5 (21.4)	33.9 (17.1)	46.2 (16.2)	37.1 (17.8)	*88.6*	*81.2*
SVA_mm_	2.5	3.6	38.2 (3.6)	33.1 (2.5)	34.9 (3.2)	29.8 (2.1)	***92.8***	*83.1*

The mean average error (MAE) are presented along with the standard deviation (SD) of each parameter evaluated in the two datasets (DS1 & DS2). The relative accuracy of prediction (Acc) is also calculated and presented in last two columns

**Fig. 4:**
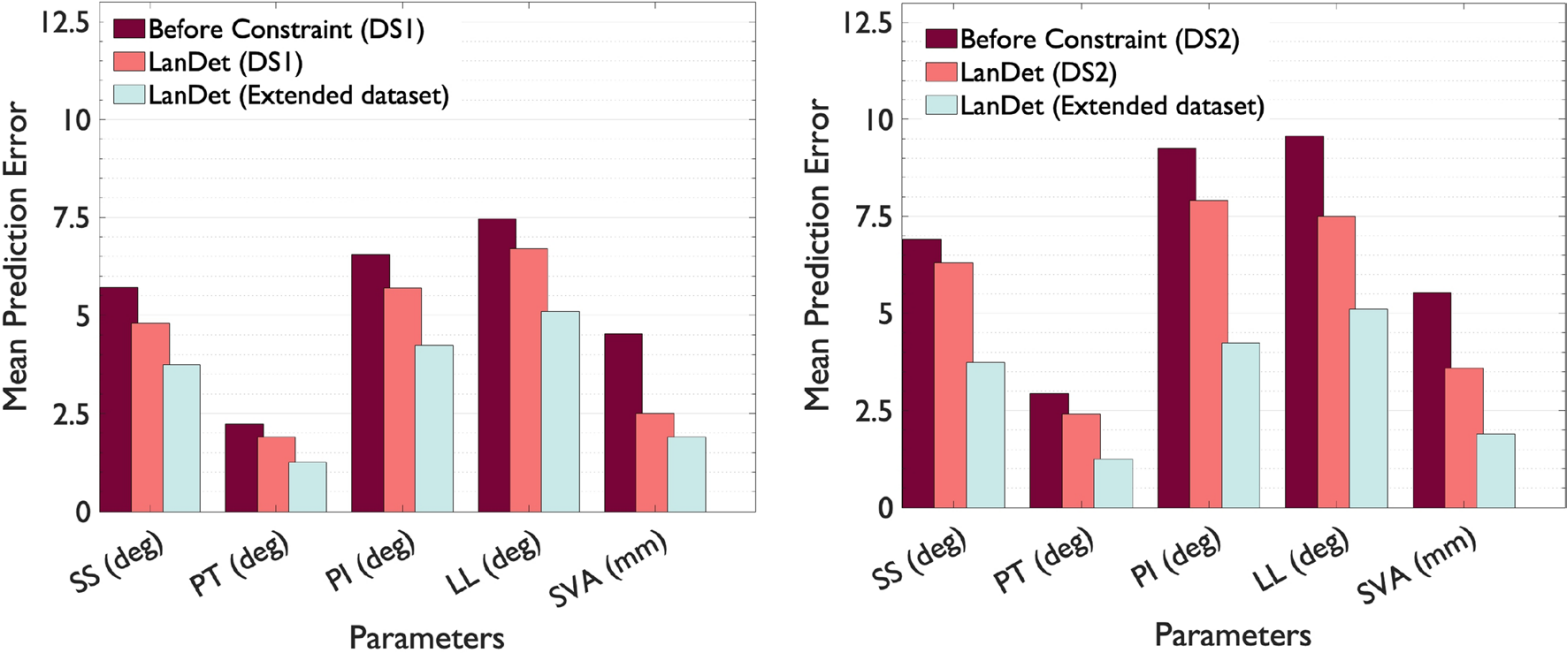
Comparison of model performance: ***LanDet*** model with physics-informed constraint vs. landmarks as objects model (before constraint) for DS1 (left) and DS2 (right). Additionally, the figure presents the performance of the ***LanDet*** model on the combined dataset, allowing for a comparison of its performance on each dataset

To highlight the impact of the physics-informed constraint approach, we compared the model’s predictions before and after applying this technique in a landmark detection as objects model using YOLOv5 algorithm, which incorporates CIoU and SIoU for enhancing bounding box prediction accuracy through geometric difference considerations (Fig. 4). As shown in the figures, the average errors for all measures decreased as a result of the model better understanding the relations between different measures through this approach.

After combining the datasets (DS1 and DS2), we evaluated our model on this combined dataset and assessed its final accuracy. For the ***LanDet*** model, evaluation revealed a mean Average Precision (*mAP*) of 0.875 at an Intersection over Union (IoU) threshold of 0.5 $(mAP_{0.5})$ across the combined dataset. Additionally, the mAP across IoU thresholds from 0.5 to 0.95, with increments of 0.05 $(mAP_{0.5:0.95})$, was recorded at 0.595. The accuracy results for SS, PT, PI, LL, and SVA were 93.1%, 94.6%, 93.4%, 91.2%, and 94.5% respectively (Table 2). Notably, the increased number of images in the training dataset and the higher quality of images in DS2 contributed to improved detection accuracy, with all exceeding 90%. Among them, PT achieved the highest accuracy of 94.6%.

**Table 2:** Statistical comparison of the values of reference manual parameter measurements (GT) and those obtained automatically by the prediction ***LanDet*** model (PR) for the combined dataset

	MAE (±SD)	Mean PR (±SD)	Mean GT (±SD)	Correlation Analysis		Acc (%)
				*R*	*p*	
**SS**^∘^	3.7 (2.7)	39.8 (9.4)	37.9 (10.3)	0.89	<0.0001	*93.1*
**PT**^∘^	1.3 (1.1)	19.2 (13.3)	20.2 (12.9)	0.98		***94.6***
**PI**^∘^	4.2 (3.1)	53.7 (12.4)	52.8 (12.3)	0.93		*93.4*
**LL**^∘^	5.1 (6.4)	38.3 (19.5)	40.3 (16.7)	0.83		*91.2*
**SVA**_mm_	2.1 (1.9)	3.5 (3.1)	3.3 (2.9)	0.96		*94.5*

The mean average error (MAE) are presented along with standard deviations (SD) of each parameter evaluated in the two datasets (DS1 & DS2). The table also include the correlation analysis using R Pearson correlation coefficients, and relative accuracy of prediction (Acc)

**Table 3:** Comparison of ***LanDet*** model estimation errors with literature reports

**Measure**	**LanDet Model**	**Literature (Reported Mean Error and SD)**	
	Mean error and SD	Upper range	Lower range
SS	$3.7 \pm 2.7^\circ$	$8.4 \pm 6.3^\circ$	$2.7 \pm 2.9^\circ$
	$R = 0.89$	$R = 0.76$	$R = 0.85$
PT	$1.3 \pm 1.1^\circ$	$2.7 \pm 2.5^\circ$	$1.2 \pm 1.7^\circ$
	$R = 0.98$	$R = 0.9$	$R = 0.98$
PI	$4.2 \pm 3.1^\circ$	$9.5 \pm 7.1^\circ$	$3.8 \pm 2.0^\circ$
	$R = 0.93$	$R = 0.72$	$R = 0.96$
LL (L5-L1)	$5.1 \pm 6.4^\circ$	$11.2 \pm 5.0^\circ$	$4.2 \pm 2.3^\circ$
	$R = 0.83$	$R = 0.79$	R = “not reported”
SVA	$2.1 \pm 1.9$ mm	$3.6 \pm 3.5$ mm	$2.0 \pm 2.4$ mm
	$R = 0.98$	$R = 0.96$	$R = 0.99$

Table 3 illustrates the comparative performance of our model against reported accuracies in the literature across various measures. The table is structured to provide a clear and concise comparison of the metrics, but it should be mentioned that the dataset used in each study is different from each other and from our datasets.

Sacrum Slope (SS): Our model predicts SS with a mean error and standard deviation of $3.7 \pm 2.7^\circ$ and a Pearson correlation coefficient (R) of 0.89. The ***LanDet*** model outperforms the lower reported accuracy range of $8.4 \pm 6.3^\circ$, $R = 0.76$ [10], demonstrating greater precision. While ***LanDet*** shows a slightly higher mean error than the upper range in the literature ($2.7 \pm 2.9^\circ$, $R = 0.85$) [26], the high correlation coefficient indicates its reliability.

Pelvic Tilt (PT): The model shows a mean error of $1.3 \pm 1.1^\circ$ with an R of 0.98 for PT. This performance is within the highest accuracy range reported in the literature, which spans from $2.7 \pm 2.5^\circ$, $R = 0.9$ [5] to $1.2 \pm 1.7^\circ$, $R = 0.98$ [13]. This indicates that our model is not only consistent with top-performing models but also tends towards a lower error margin, highlighting its precision.

Pelvic Incidence (PI): For PI, our model registers $4.2 \pm 3.1^\circ$, $R = 0.93$, compared to a reported range in the literature of $9.5 \pm 7.1^\circ$, $R = 0.72$ [10] to $3.8 \pm 2.0^\circ$, $R = 0.96$ [26]. Here, our model demonstrates significant accuracy, surpassing the lower range of reported values and closely matching the higher accuracy models in both precision and correlation.

For the Lumbar Lordosis (LL) measure, our model’s errpr is represented as $5.1 \pm 6.4^\circ$ with a Pearson correlation coefficient (R) of 0.83. When compared with the reported accuracies in the literature, our model demonstrates competitive performance. The lower range of accuracy in the literature is reported as $11.2 \pm 5.0^\circ$, $R = 0.79$ [10], while the upper range is $4.2 \pm 2.3^\circ$ [11], with the correlation coefficient not reported.

Sagittal Vertical Axis (SVA): Our model estimates SVA with a mean error of $2.1 \pm 1.9$ mm and a correlation coefficient of 0.98. This compares favorably with the literature range of $3.6 \pm 3.5$ mm, $R = 0.96$ [12] to $2.0 \pm 2.4$ mm, $R = 0.99$ [13]. Our model’s results for SVA are within the range of existing models and demonstrate a balance between accuracy and reliability.

In summary, our model exhibits robust performance across a range of measures, consistently matching or surpassing the literature-reported accuracy. There are some measures for which our model accuracy is less than that reported in the literature. [13, 26]. This may be because the dataset size, image quality, and number of detection classes are different in each study including ours. For instance, [13] had a dataset including  2500 images compared to our dataset of  1400 images. Furthermore, [26] mentioned that cases involving minors whose skeletons have not fully matured and instances of degenerative disk disease, where the vertebra corners are challenging to discern, were omitted from their dataset. Additionally, only high-quality images with appropriate contrast and brightness levels for clear observation were included, while we excluded very few from our dataset to enhanse its capability for a real-world application.

### Limitations and advantages

**Fig. 5:**
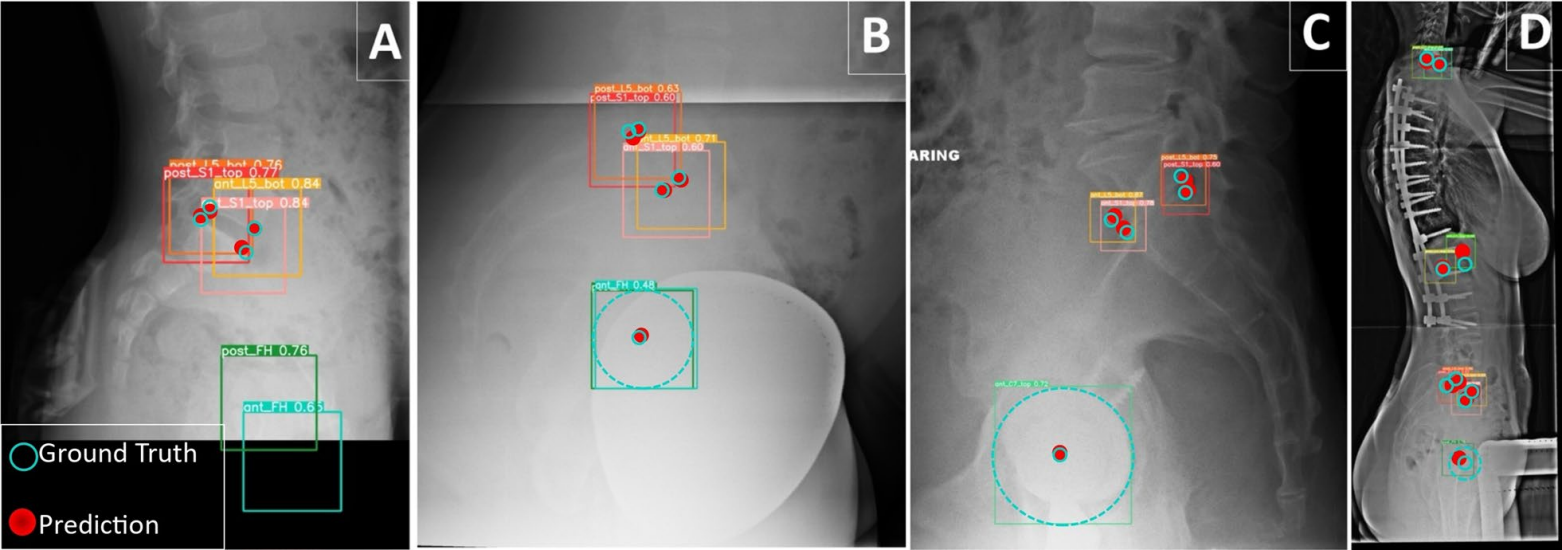
Challenging cases successfully addressed by the model in the datasets: **A** Partially cut-off images, **B** Images with obstacles in the hip region, **C** Images from patients with hip implants, **D** Images from patients with spinal implants

To The model performance compares favorably with the literature and exhibits unique capabilities in handling special cases and overcoming challenges related to adjacent landmark identification. We evaluated the model on two distinct datasets, demonstrating its adaptability to different scenarios and images from diverse sources. Approximately 5% of the datasets comprised images of low quality or partial visibility, while around 15% contained images with hip or spine implants. Figure 5 showcases four specific instances where the model accurately identified the required landmarks, even with patients having spinal or hip implants or images with partial cutoff or obstructed by protective shields. Despite the scarcity of training images for such special cases, our model proved successful in handling them effectively. It is worth noting that manually annotating landmarks in such cases, particularly those with obstacles or partial image cutoff, can be challenging.

**Fig. 6:**
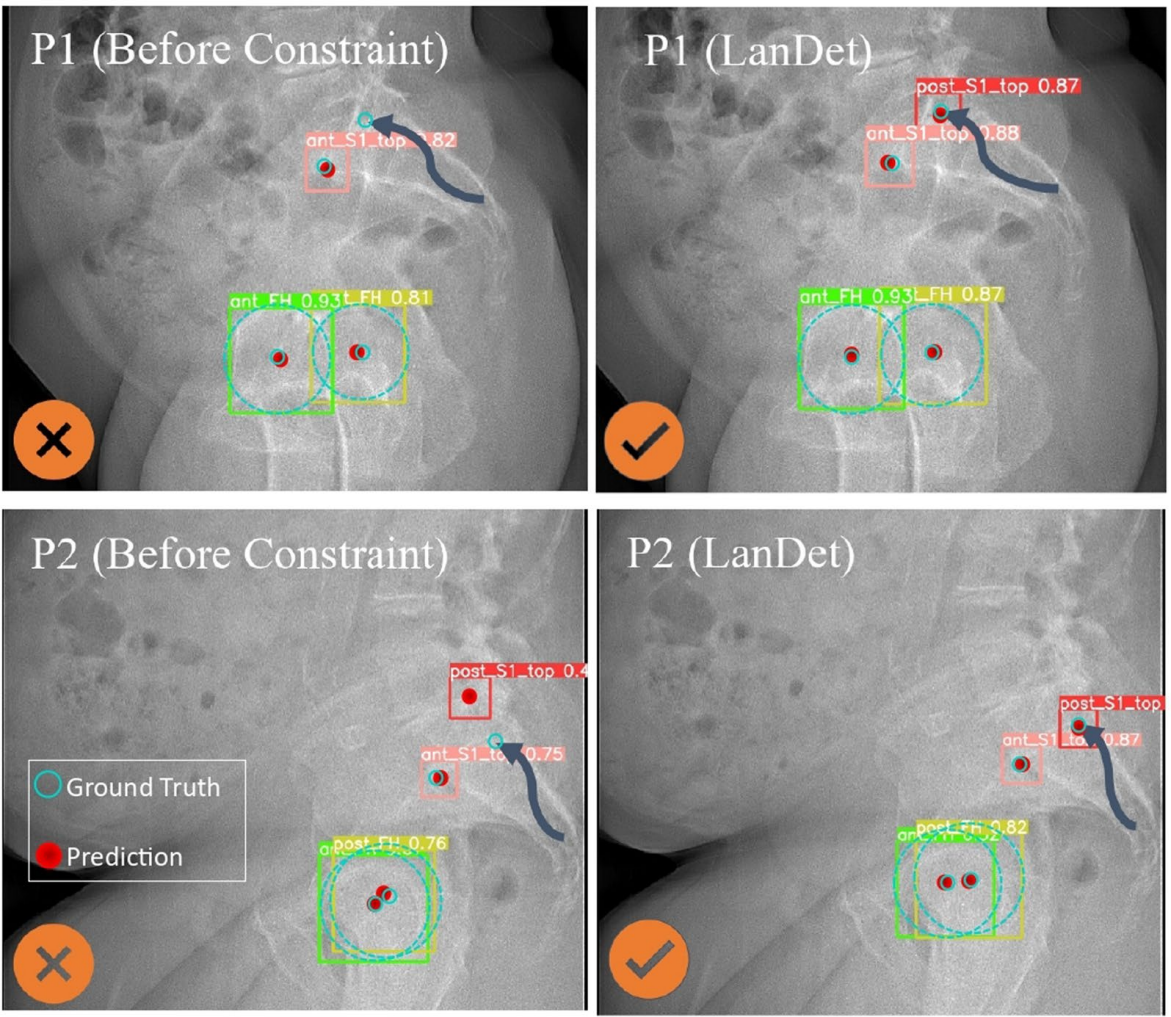
The advantage of applying physics-informed constraints, which improved the accuracy of localization of some similar and adjacent landmarks

Previous studies in the literature [5, 13] have highlighted the challenge of missing specific landmarks and difficulty in distinguishing between adjacent and similar anatomical landmarks. In our research, we encountered similar issues until we introduced physics-informed constraints into our model. As illustrated in Fig. 6, the ***LanDet*** model successfully addresses these challenges by incorporating the concept of landmarks as objects with the integration of physics-informed constraints.

The primary limitation that deserves discussion is the availability of data. As expected, a larger training dataset would lead to better model performance. While the model demonstrated robustness in detecting landmarks in challenging cases, improving the accuracy of these detections would be possible by increasing the number of images from patients with abnormalities or spinal implants in the dataset. Furthermore, it is essential to highlight that our model did not undergo external validation, meaning it was not tested on images from different medical centers. External validation would provide valuable insights into the model’s generalizability and effectiveness in different clinical settings.

### Model reliability and level of agreement between the model and surgeons

**Fig. 7:**
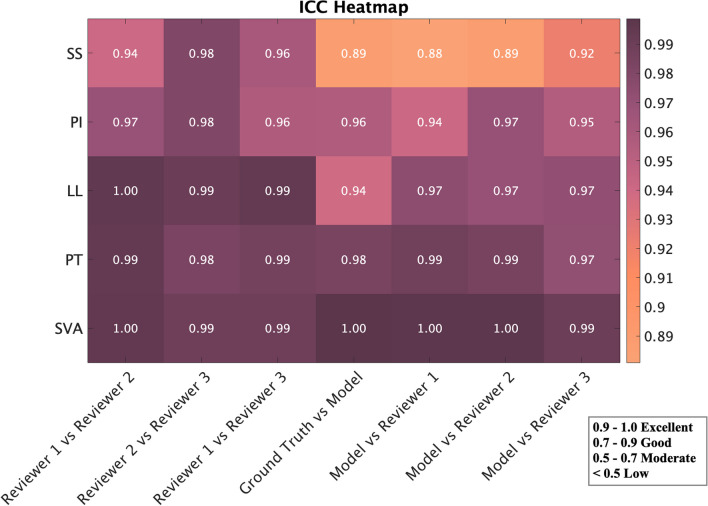
ICC metric results for the evaluation of model reliability by comparing the extracted results from the Model with Ground Truth, and three surgeons (Reviewer1: senior spine surgeon, Reviewer2: senior neurosurgeon, and Reviewer3: senior orthopedic surgeon)

We conducted a comprehensive evaluation of our model’s reliability and performance in comparison to expert surgeons. Our assessment involved comparing the extracted metrics from the model against those obtained from three separate surgeon annotations. To quantify the degree of agreement, we employed the ICC metric, the results of which are depicted in Fig. 7. Our model exhibited a notably higher consistency with the surgeons, surpassing previously reported levels in the literature [13].

The ***LanDet*** deep learning architecture demonstrated exceptional reliability across all measured parameters, effectively matching the precision of assessments conducted by the surgeon reviewers. Our findings strongly corroborate prior research conclusions, which consistently highlighted increased error rates, variability, and reduced consistency when measuring SS [5, 13, 27].

## Conclusion

In this paper, we presented a novel deep learning approach for detecting anatomical landmarks as objects, surpassing the limitations of previous models that mostly relied on heat-map regression. By incorporating physics-informed constraints into our deep learning models, we achieved competitive results in landmark detection accuracy. Moreover, our approach demonstrated robustness in challenging scenarios, including cases with implants, protected regions, and partially obscured images, even when training data for such scenarios was limited. Furthermore, our model effectively addressed the issue of mis-detection of similar or adjacent landmarks. The landmark detection performance for SS, PT, PI, LL, and SVA measures was evaluated, comparing results between datasets of different sizes and against the existing literature. Our model achieved competitive performance while offering the aforementioned advantages. To assess the reliability of our model, we compared its predictions against those of three senior surgeons, using the ICC metric. The results revealed a high level of agreement between our model and the expert surgeons.

In conclusion, our proposed deep learning approach presents an efficient approach in the field of anatomical landmark detection. Its success in handling challenging scenarios and achieving comparable performance to expert evaluations makes it a valuable tool for clinical applications and research studies. The integration of physics-informed constraints into the deep learning framework opens new possibilities for accurate and robust landmark detection in medical imaging.
